# A comprehensive assessment of resting state networks: bidirectional modification of functional integrity in cerebro-cerebellar networks in dementia

**DOI:** 10.3389/fnins.2014.00223

**Published:** 2014-07-30

**Authors:** Gloria Castellazzi, Fulvia Palesi, Stefano Casali, Paolo Vitali, Elena Sinforiani, Claudia A. M. Wheeler-Kingshott, Egidio D'Angelo

**Affiliations:** ^1^Department of Industrial and Information Engineering, University of PaviaPavia, Italy; ^2^Brain Connectivity Center, C. Mondino National Neurological InstitutePavia, Italy; ^3^Department of Physics, University of PaviaPavia, Italy; ^4^Department of Brain and Behavioral Sciences, University of PaviaPavia, Italy; ^5^Brain MRI 3T Mondino Research Center, C. Mondino National Neurological InstitutePavia, Italy; ^6^Neurology Unit, C. Mondino National Neurological InstitutePavia, Italy; ^7^NMR Research Unit, Department of Neuroinflammation, Queen Square MS Centre, UCL Institute of NeurologyLondon, UK

**Keywords:** Alzheimer disease, mild cognitive impairment, resting state fMRI, functional connectivity alterations, cerebro-cerebellar networks

## Abstract

In resting state fMRI (rs-fMRI), only functional connectivity (FC) reductions in the default mode network (DMN) are normally reported as a biomarker for Alzheimer's disease (AD). In this investigation we have developed a comprehensive strategy to characterize the FC changes occurring in multiple networks and applied it in a pilot study of subjects with AD and Mild Cognitive Impairment (MCI), compared to healthy controls (HC). Resting state networks (RSNs) were studied in 14 AD (70 ± 6 years), 12 MCI (74 ± 6 years), and 16 HC (69 ± 5 years). RSN alterations were present in almost all the 15 recognized RSNs; overall, 474 voxels presented a *reduced* FC in MCI and 1244 in AD while 1627 voxels showed an *increased* FC in MCI and 1711 in AD. The RSNs were then ranked according to the magnitude and extension of FC changes (*gFC*), putting in evidence 6 RSNs with prominent changes: DMN, frontal cortical network (FCN), lateral visual network (LVN), basal ganglia network (BGN), cerebellar network (CBLN), and the anterior insula network (AIN). Nodes, or hubs, showing alterations common to more than one RSN were mostly localized within the prefrontal cortex and the mesial-temporal cortex. The cerebellum showed a unique behavior where voxels of decreased *gFC* were only found in AD while a significant *gFC* increase was only found in MCI. The *gFC* alterations showed strong correlations (*p* < 0.001) with psychological scores, in particular Mini-Mental State Examination (MMSE) and attention/memory tasks. In conclusion, this analysis revealed that the DMN was affected by remarkable FC increases, that FC alterations extended over several RSNs, that derangement of functional relationships between multiple areas occurred already in the early stages of dementia. These results warrant future work to verify whether these represent compensatory mechanisms that exploit a pre-existing neural reserve through plasticity, which evolve in a state of lack of connectivity between different networks with the worsening of the pathology.

## Introduction

Alzheimer's disease (AD) is a progressive neurodegenerative disorder characterized by diffuse cortical atrophy, most pronounced in the mesial-temporal lobe (Sluimer et al., 2009; Ferreira et al., 2011). The pathological hallmarks of AD are the accumulation of β amyloid (Aβ_1–42_) plaques and tau tangles concentrated in the prefrontal and mesial-temporal lobe (Blennow et al., 2006; Holtzman et al., 2011; Serrano-Pozo et al., 2011; Herskovits et al., 2013). Mild cognitive impairment (MCI) is a less sever clinical state that can evolve in AD but demonstrate less pronounced brain atrophy. It is possible that, during the progression from MCI to AD, a complex rearrangement occurs in brain networks. For example, PET-MRI studies have shown that, in MCI, hypometabolism is predominant in precuneus/posterior cingulum, (Morbelli et al., 2010), while amyloid deposition is more conspicuous in prefrontal lobe (La Joie et al., 2012). These findings suggest a dissociation between hystopathological and functional changes, which can extend over brain regions not affected by neurodegeneration and may differ in nature and localization at different stages of the pathology.

Functional magnetic resonance imaging (fMRI) is a leading candidate for assessing changes in functional connectivity (FC), suitable to study MCI and AD thanks to the short acquisition protocol without the need of performing a task. By measuring FC between spatially distinct brain regions, resting-state fMRI (rs-fMRI) allows the identification of several networks (RSNs) (Calhoun et al., 2001; Beckmann et al., 2005; Damoiseaux et al., 2006, 2008; Cole et al., 2010), in which separate brain areas show MR signal correlations in the absence of any specific external stimulation (Biswal et al., 1995; Cordes et al., 2001; Greicius et al., 2003; Fox and Raichle, 2007; Zhang and Raichle, 2010). Because of the striking overlap between areas of amyloid deposition and areas involved in the default mode network (DMN) (Mormino et al., 2011), most rs-fMRI studies in AD patients have focused the attention on alterations of the DMN (Jones et al., 2011; Petrella et al., 2011). The DMN is active during episodic and autobiographical memory retrieval and shows decreased activity during cognitive tasks demanding attention to external stimuli (Raichle et al., 2001; Greicius et al., 2003, 2004; Wu et al., 2011; Brier et al., 2012; Koch et al., 2012). In AD, the DMN has shown decreased FC in the precuneus and posterior cingulate cortex, revealing a good matching between reduced FC, cortical atrophy and memory impairment (Binnewijzend et al., 2012). Only a few studies suggested also the involvement of other RSNs, including visual cortex, basal ganglia and cerebellum (Agosta et al., 2012; Binnewijzend et al., 2012; Brier et al., 2012). Interestingly, the cerebellum is a common area that can be thought as a node of several RSNs including the DMN. Although previous works have suggested a cerebellar involvement in AD and MCI (Wang et al., 2007; Kaufmann et al., 2008; Thomann et al., 2008; Bai et al., 2009, 2011b; Solé-Padullés et al., 2009; Teipel et al., 2013), the alteration in FC in the cerebellum in AD and MCI remains unclear.

In this work we have characterized the changes occurring in multiple RSNs in two groups of patients, with clinical diagnosis of MCI and AD, compared to healthy subjects. In order to take into consideration positive and negative alterations in terms of both FC amplitude and voxels extension, we introduced a generalized FC (*gFC*) parameter. Then we ranked the networks and identified key patterns when comparing changes between MCI and AD and HC and we investigated the presence of hubs of alterations (nodes) at the intersection of multiple networks. We finally assessed clinical correlations and characteristics that could be useful in future longitudinal studies aimed at addressing the issue of predicting clinical conversion.

## Methods

### Subjects

A total of 56 subjects (mean age 70 ± 6) were recruited for this study among those suffering from subjective or objective memory complaint, attending regularly the Memory Clinic of the Neurological Institute C. Mondino, Pavia, Italy. Subjects were selected based on their neuropsychological examination using a standardized battery of tests (see section below), which evaluated different cognitive domains (Spinnler and Dall'ora, 1987). Exclusion criteria were: age >80 years, significant medical, neurological (different from AD) psychiatric disease as well as significant cerebrovascular disease (Hachinski et al., 1975; Binnewijzend et al., 2012). In detail, patients with significant Central Nervous System (CNS) disorders other than AD (e.g., Parkinson's disease and other extra-pyramidal disorders, multiple sclerosis, epilepsy, significant focal or vascular intracranial pathology, clinical evidence of cerebrovascular accident, and/or previous head injury with loss of consciousness) were excluded. Written informed consent was provided by all the subjects or their lawful caregiver. Fourteen patients were excluded because of data quality, mainly motion. Some images had such artifacts that were excluded on visual inspection. When in doubt, realignment parameters were also considered and data excluded if the subject had a maximum displacement in any of the cardinal directions (x, y, z) that was larger than 3 mm, or a maximum spin (x, y, z) larger than 3°. After inspection, 42 subjects of the original 56 were included in this study. Based on the clinical evaluation, subjects were divided into two groups: 14 patients (8 females, mean age 74 ± 6) were classified as AD (NINCDS2-ARDAcriteria) (McKhann et al., 2011) and 12 patients (10 females, mean age 70 ± 6) as MCI (Petersen et al., 2001, 2009; Petersen, 2009). MCI subjects were classified as having amnestic MCI (a-MCI) if the memory was the only impaired domain, multi-domain MCI (md-MCI) if the impairment was not limited to the memory. Based on this rule, 3 of the 12 MCI subjects recruited for this study were classified as a-MCI, while the remaining 9 patients were md-MCI. Because of the small sample of a-MCI, all the MCI subjects (both a-MCI and md-MCI) were combined in a unique group. In order to obtain a reference metric for our findings, 16HC (10 females, mean age 69 ± 5) were recruited on a volunteer base through a local recreational association (“Argento Vivo,” Bereguardo, PV) and underwent the MRI examination.

### Clinical and neuropsychological examination

Subjects underwent clinical and neuropsychological testing, which evaluated different cognitive domains (Spinnler and Dall'ora, 1987; Spinnler and Tognoni, 1987) including: Mini-Mental State Examination (MMSE) (Folstein et al., 1975), trial making test part A and B (TMT-A and TMT-B) (Reitan, 1958), memory for prose (MP) (Novelli et al., 1986), category fluency (CF), and semantic fluency (SF) (Randolph et al., 1993), Rey complex figure copy test (ROCF-copy) and Rey complex figure recall (ROCF-rec) (Osterrieth, 1944; Caffarra et al., 2002). For each test age-, gender-, and education-corrected scores were calculated from the raw scores. Corrected scores were then transformed in equivalent scores ranging from 0 (pathological) to 1 (lower limit of normal) and 2–4 (normal). Only corrected scores were used in the statistical analysis.

### MRI acquisitions

All data were acquired using a 1.5T MR Philips Intera Gyroscan (Philips Healthcare, Best, The Netherlands) with an 8-channel head (SENSE) third-party coil. For each subject a fast field echo-planar imaging (FFE-EPI) protocol was acquired for rs-fMRI with *TR*/*TE* = 3000/60 ms, voxel size = 2.2 × 2.2 × 4 mm^3^, FOV = 250 × 250 mm^2^, 26 slices, SENSE factor = 3.1, 100 repeated volumes. For anatomical reference a volumetric 3DT1-weighted acquisition was also collected using a fast field echo (FFE) sequence (*TR*/*TE* = 8.6/4 ms; flip angle 8°; 170 sagittal slices; slice thickness = 1.2 mm; FOV = 240 mm; acquisition matrix = 192 × 192, reconstructed to 256 × 256; in-plane resolution 1.25 × 1.25 mm^2^, reconstructed to 0.94 × 0.94 mm^2^).

All the MRI analysis was performed on a workstation with Linux Ubuntu 12.04, running SPM8 (Wellcome Department of Cognitive Neurology, http://www.fil.ion.ucl.ac.uk/), Matlab R2009a (The MathWorks, Natick, Mass, USA http://www.mathworks.com/) and FSL (FMRIB Software Library, version 4.1.9, http://www.fmrib.ox.ac.uk/fsl/).

### fMRI analysis

For each recruited subject rs-fMRI images were analyzed using Independent Component Analysis (ICA) to characterize RSNs. ICA results were analyzed using the Multivariate Exploratory Linear Optimized Decomposition into Independent Components (MELODIC) method as implemented in FSL.(Beckmann et al., 2005)

#### Data pre-processing

Individual subject's pre-processing consisted in motion correction, brain extraction, spatial smoothing using a Gaussian kernel of full-width-at-half-maximum (FWHM) of 6 mm, and high pass temporal filtering equivalent to 150 s (0.007 Hz). rs-fMRI volumes were then registered to the individual's structural 3DT1 scan using FMRIB's Linear Image Registration Tool (FLIRT) and subsequently to standard space (MNI152) using FMRIB's Nonlinear Image Registration Tool (FNIRT) with default options.

#### Independent component analysis (ICA)—identification of RSNs

Pre-processed functional data, containing 100 time points (volumes) for each subject, were temporally concatenated across subjects to create a single 4-dimensional data set. The dataset was decomposed into independent components (ICs), with an automatic estimation for the number of components, which resulted in spatial maps, each with an associated time course. Model order was estimated using the Laplace approximation to the Bayesian evidence for a probabilistic principal component model. Some of the ICs were identified as noise while others as RSNs, based on previous literature (Beckmann et al., 2005; Damoiseaux et al., 2006; Smith et al., 2009; Cole et al., 2010). This method is run on the entire dataset (i.e., the selected 42 subjects) and decomposes data into spatial maps that are the ICs relative to the total processed dataset, or the multi-subject ICA components. This means that ICs are the same for each subject and represent the maps within which inference between groups (AD, MCI, and HC) is then evaluated applying dual regression.

#### Dual regression—evaluation of group differences within the RSNs

A non-parametric permutation test, referred to as “dual regression” technique, was then applied to compare group-specific FC maps for each independent spatial component. In particular with this analysis differences between HC, MCI, and AD groups were tested using 4 different comparisons, which we will refer to as *contrasts* (MCI < HC; AD < HC; MCI > HC; and AD > HC). Dual regression allows for between-subjects analysis by carrying out a voxel-wise comparisons of the resting FC (Filippini et al., 2009). In this study the dual regression analysis was carried out on the total ICs using age, gender and education level as additional covariates in the permutation tests (http://fsl.fmrib.ox.ac.uk/fsl/fslwiki/DualRegression).

In detail, spatial ICs were used in a linear model fit against each individual rs-fMRI data set (spatial regression), to create matrices that described the temporal dynamics for each component and subject separately. These matrices were used in a linear model fit against the associated subject's rs-fMRI data set (temporal regression), to estimate subject-specific spatial correlation maps. Subsequently spatial maps of all subjects were collected into single 4-dimensional files for each original independent component and tested voxel-wise for statistically significant differences between groups using nonparametric permutation tests (10,000 permutations) (Filippini et al., 2009; Binnewijzend et al., 2012). The resulting statistical maps were family-wise error (FWE) corrected for multiple comparisons using the threshold-free cluster enhancement (TFCE) method. In detail, TFCE uses the raw statistic maps produced in the initial steps of the dual regression methods and yields images, in which the voxel-wise values represent the amount of cluster-like local spatial support (Smith and Nichols, 2009). Voxels that survived a statistical threshold of *p* ≤ 0.05 were considered significant (Binnewijzend et al., 2012) and were saved as tstat_FC_ maps.

#### Global network analysis—ranking of RSN alterations

In order to study FC changes within each RSN and to establish a ranking of the networks in terms of their alterations, for each *contrast* we defined a global parameter taking into account (1) the extension of the clusters and (2) the magnitude of the FC changes. For each RSN, we considered (1) the number of altered voxels in the tstat_FC_ map (*N*_*tstatFC*_) with respect to the total number of voxels of the RSN itself (*N*_*RSN*_). For each RSN we calculated (2) the overall FC change of the group (*meanFC*) by firstly averaging each subject's tstat_FC_ map and then evaluating its mean for each group to give *meanFC*_*HC*_, for the HC, and *meanFC*_*P*_, for patients, with p = MCI or AD. We then combined these parameters in a new index, called *global FC* (*gFC*) *index* as follows:

(1) $$gFC=\left|\left(\frac{meanFC_{HC}-meanFC_{P}}{meanFC_{HC}}\right)\right|\;\cdot \;\frac{N_{tstatFC}}{N_{RSN}}$$

For each contrast, we were therefore able to rank the RSN alterations in terms of their decreasing *gFC* values.

#### Individual network analysis—regional quantification of RSN alterations

To assess spatial distributions of RSNs changes we divided the brain according to cortical regions (Brodmann, 2006; Diedrichsen et al., 2009) into seven different areas, (*l*): cerebellum (C), prefrontal (PreF), precentral (PreC), limbic (L), occipital (O), parietal (P), and temporal (T). In particular, PreF includes the inferior, middle and superior frontal cortices. Each of the seven anatomical regions has been obtained grouping different Brodmann areas on anatomical and functional basis (see Table 1 for details). The temporal (T) sub-area includes the lateral (inferior, middle, and superior temporal gyri), inferior (fusiform gyrus), and mesial (amygdala, parahippocampal gyrus, and hippocampus) temporal cortices. Similarly to the evaluation of the *gFC index*, for each *contrast* we considered both the *FC* changes and the extension of such changes in each areas *l*. We used the *tstats*_*FC*_ maps to quantify the mean value of altered *FC* within each RSN within each sub-area [*meanFC*(l)], and calculated the total number of altered voxels [*N*_*tstatFC*_(l)] for the same RSN. We defined relative changes compared to the overall *meanFC*(l)_*HC*_ in *HC* and the total number of voxels in the considered RSN, *N*_*RSN*_. For each sub-area, *l*, and for each *RSN* we then combined these parameters into a regional global index, r*gFC*(l) as follows:

(2) $$\text{r}gFC\left(l\right)=\left|\left(\frac{meanFC{\left(l\right)}_{HC}-meanFC{\left(l\right)}_{P}}{meanFC{\left(l\right)}_{HC}}\right)\right|\;\cdot \;\frac{N_{tstatFC}\left(l\right)}{N_{RSN}}$$

**Table 1:** **Brain subdivision used to assess the spatial distributions of RSN changes**.

**Area**	**Anatomy**	**Function**	**Anatomical location**	**Brodmann area**
PreF	Prefrontal cortex	Associative	Dorso lateral prefrontal cortex	BA 9
				BA 46
			Orbito frontal cortex	BA 10
				BA 11
				BA 47
PreC	Precentral cortex	Visual	Frontal eye field	BA 8
		Prim motor	Motor cortex	BA 4
		Pre/Suppl motor		BA 6
		Primary language	Broca's area	BA 44
				BA 45
P	Parietal cortex	Mixed	Angular gyrus	BA 39
			Supramarginal	BA 40
		Associative	Gustatory cortex	BA 43
			Somatosensory association cortex	BA 5
				BA 7
		Primary somato sensory	Primary somato-sensory cortex	BA 1
				BA 2
				BA 3
T	Temporal cortex	Mixed	Superior temporal gyrus	BA 22
				BA 38
		Associative	Retrosubicular	BA 48
			Inferior temporal	BA 20
			Medial temporal	BA 21
			Amygdala	BA 25
			Hippocampus	BA 28
			Para hippocampal gyrus	BA 34
				BA 35
				BA 36
			Piriform cortex	BA 27
			Fusiform gyrus	BA 37
		Primary auditory	Auditory cortex	BA 41
				BA 42
O	Occipital cortex	Visual	Visual cortex	BA 17
				BA 18
				BA 19
L	Limbic	Associative	Post cingulate	BA 23
			Anter cingulate	BA 24
				BA 32
			Retrospl cingulate	BA 29
			Cingulate	BA 30
			Ectosplenial	BA 26
C	Cerebellum	Various sensory-motor and cognitive functions		

#### Definition of nodes

On the basis of the previous mentioned parcellation of the brain into seven different sub-regions (C, PreF, PreC, L, O, P, and T), we considered as nodes or hubs those sub-regions in which multiple networks presented coexistent functional alteration. For each node we were able to calculate the percentage of functional alteration calculating the ratio between the total number of altered voxels within the node and the total number of voxels that fall at least within a RSN.

### Voxel based morphometry (VBM) analysis

To account for possible interactions between structural differences as measured by atrophy and the observed FC differences between AD, MCI, and HC, we performed Voxel Based Morphometry (VBM) analysis on the 3DT1-weighted images, using SPM8 following a standard VBM protocol (Testa et al., 2004; Boccardi et al., 2005; Frisoni et al., 2005; Mechelli et al., 2005; Pennanen et al., 2005; Filippini et al., 2006). VBM analysis was performed on all 42 subjects included in the present study [14 AD patients, 12 MCI, and 16 healthy controls (HC)].

Briefly, probability maps were computed for gray matter (GM), white matter (WM), and cerebro-spinal fluid (CSF) (Good et al., 2002). Following these steps: for each subject 3D T1-weighted images were normalized to a high resolution T1 MNI152 template through affine and nonlinear transformations. The resulted image was then segmented into GM, WM, and CSF using the customized priors, masked to remove non-brain tissue voxels, modulated, and finally smoothed with a 8 mm Gaussian kernel (Ashburner and Friston, 2001).

Statistical analysis was performed on each tissue priors using the general linear model (GLM) framework and the resulting t-statistic maps were thresholded at *p* < 0.001. In order to reduce the number of false positives we set an extent threshold of 30 voxels (Ashburner and Friston, 2001; Mechelli et al., 2005).

In order to verify to what extent FC changes are co-localized with VBM changes, in particular within the DMN we: (1) calculated the overlap between voxels in the DMN with those showing the GM atrophy; (2) we calculated the overlap between the FC decrease in the DMN and voxels affected by GM atrophy.

### Effects of cortical atrophy on FC changes

As a sensitivity analysis, to verify whether cortical volume influenced the fMRI results and in particular the FC changes, a second dual-regression analysis was carried out on all the ICs using the relative GM volume, expressed as ratio between GM absolute volume (mm^3^) and intracranial volume (mm^3^) as a further additional covariate in the permutation tests (Oakes et al., 2007; Damoiseaux et al., 2008; Binnewijzend et al., 2012).

### Non-imaging statistics

All other statistical analyses were performed using SPSS (version 17.0; SPSS, Chicago, IL, USA). For continuous measures, differences between groups were assessed using one-way analysis of variance (ANOVA) with *post-hoc* Tamhane tests to correct for multiple comparisons. Data of TMT-A, ROCF-copy, and ROCF-rec were log transformed as Kolmogorov-Smirnov tests showed they were not normally distributed. We performed a χ^2^ test to compare frequency distributions of age, gender and education level. We performed Pearson's correlation analysis to assess correlations between regional FC values (r*gFC* index) within the RSNs (extracted mean *z*-values from the clusters of voxels showing regional FC differences) and neuropsychological test results. In order to facilitate the understanding of all the statistical comparisons between r*gFC* and neuropsychological scores we performed the correlation analysis considering the inverse of TMT-A (1/TMT-A) and of the TMT-B (1/TMT-B) so that we obtained the same trend, i.e., the lower the score the more severe the impairment. A very strict statistical threshold of *p* < 0.001 was considered for studying the correlation between the r*gFC* index and neuropsychological data.

## Results

In this rs-fMRI investigation, 15 RSNs were identified. RSN alterations in MCI and AD compared to HC were observed in several networks and were ranked taking into account FC changes (both increased and decreased) as well as voxel extension changes, according to the *gFC* parameter (see Methods). This strategy put into evidence 6 RSNs that were prominently involved with different patterns in groups of subjects with different stages of dementia. For each of these 6 RSNs we also extensively analyzed FC changes based on the r*gFC* index to assess the regional involvement of different cortical areas. A comprehensive analysis of these changes is presented in the sections below. All the changes reported in this study were significant at *p* < 0.05, TFCE-corrected.

### Comparison of subject's populations

No differences in age, sex or level of education distinguished the AD, MCI and HC groups. However, as expected from the pathological state of MCI and AD, differences in neuropsychological tests were observed between themselves and HC (Table 2). Multiple ANOVA revealed significantly different scores between the groups (*p* < 0.001) for the MMSE examining cognitive impairment, the TMT examining visual attention and task switching, the MP examining episodic memory, the RPCF-rec examining visuospatial construction, visuographic memory as well as planning and executive functions on recall. However, no differences in ROCF-copy index, were observed (*p* = 0.133). By considering the specific ANOVA subgroups, AD were worse than either MCI or HC in all neuropsychological tests on cognitive performance (*MMSE, TMT, MP, ROCF-rec*), while MCI were worse than HC only in a limited set of tests on memory and attention (*MP* and *TMT*).

**Table 2:** **Demographics and neuropsychological ANOVA test between groups**.

	**HC subjects (*n* = 16)**		**MCI subjects (*n* = 12)**		**AD subjects (*n* = 14)**		***p*-value**
	**mean**	***SD***	**mean**	***SD***	**mean**	***SD***	
Age	69	5.13	73.58	6.18	70.36	5.56	0.108
Sex (% female)	75	0.44	67	0.49	71	0.46	0.897
Education	8.56	4.42	7.92	4.27	7.43	2.76	0.729
MMSE^a^	28.13	1.43	24.64	2.03	20.70	2.71	<0.001^*^
TMT-A^a^^,^^c^	47.63	12.04	78.55	22.83	112.92	43.78	<0.001^*^
TMT-B^b^^,^^c^	102.31	40.69	147.82	121.60	336.43	281.70	0.004^*^
MP^b^	11.88	1.75	8.35	4.22	5.45	2.52	<0.001^*^
ROCF-copy^a^	33.84	2.04	32.40	7.44	29.77	5.76	0.133
ROCF-rec^b^	14.22	2.73	13.83	4.00	7.17	5.69	<0.001^*^
CF	14.60	2.26	14.25	3.99	11.27	2.73	0.009^*^
SF^b^	28.77	3.09	27.95	7.40	20.50	6.06	0.003^*^

*The table reports, for the AD, MCI, and HC groups, the score values (mean and SD) for different neuropsychological test comprising the mini mental state examination (MMSE), trial making test part A and B (TMT-A and TMT-B), memory for prose (MP), category fluency (CF) and semantic fluency (SF), Rey complex figure copy test (ROCF-copy) and Rey complex figure recall (ROCF-rec). p-values show statistically significant differences between the HC, MCI and AD groups*.

* *p* < 0.01 *threshold for statistically significant difference between groups*.

a *Missing data of 1–2 subjects*.

b *Missing data of 3–10 subjects*.

c *Lower scores indicate better (faster) performance*.

### Resting state networks identification

All subjects were scanned for rs-fMRI and the data were analyzed with ICA (see Methods for details). This processing resulted in 41 independent components, 23 of which were recognized as part of 15 full RSNs based on their frequency spectra and spatial patterns (Beckmann et al., 2005; Damoiseaux et al., 2006; Smith et al., 2009; Cole et al., 2010). This means that, out of the 23 ICs that were not cataloged as noise, 8 ICs were not full RSNs, but portions of the remaining 15 full RSNs whose frequency patterns was altered by noise and were therefore discarded. The remaining 18 components, that made up the 41 found by ICA, reflected artifacts like movement, physiological noise or CSF partial volume effect (Gour et al., 2014).

**Figure 1:**
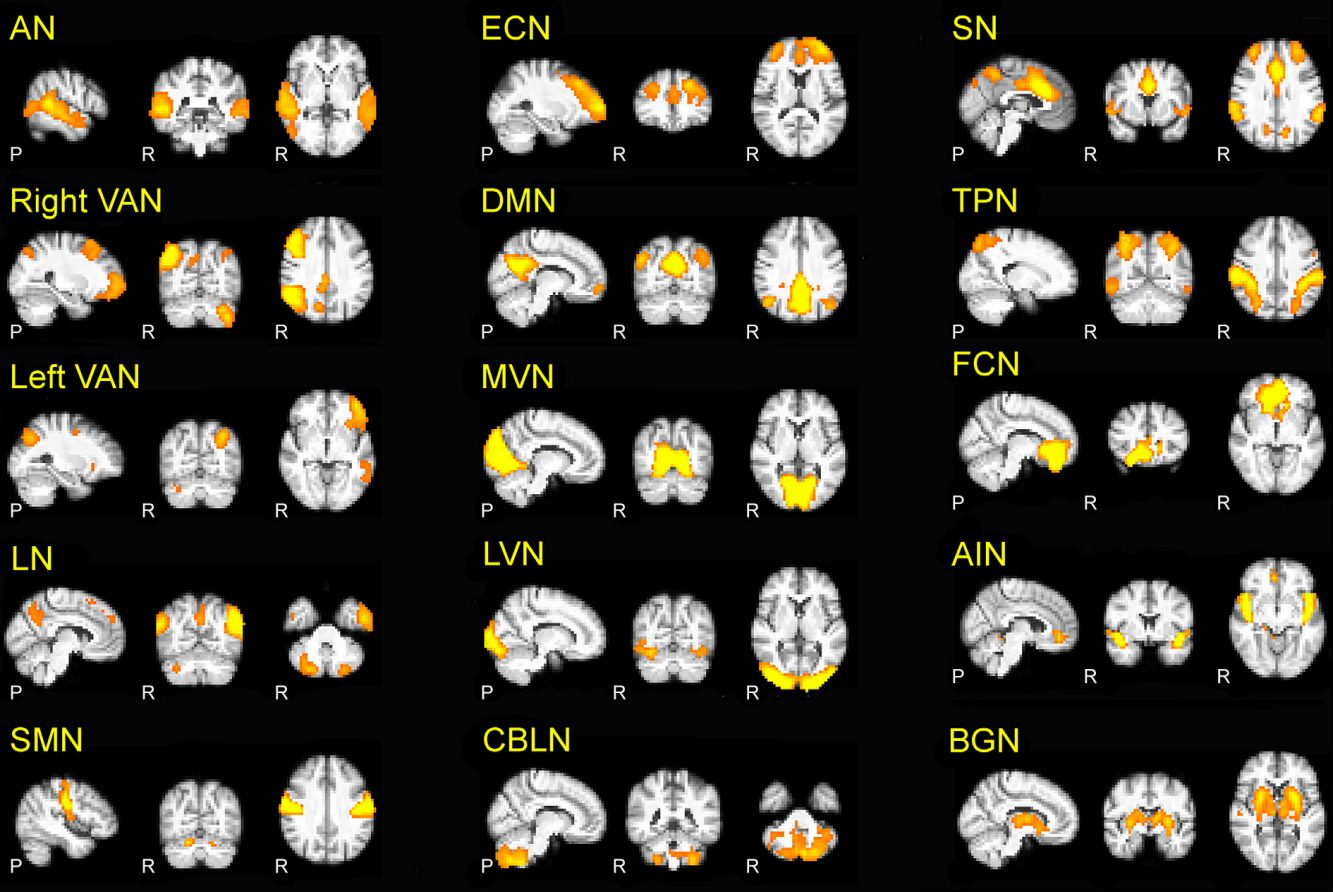
**The 15 RSNs identified in the present investigation**. Auditory network (AN), right and left ventral attention networks (VAN), language network (LN), sensory-motor network (SMN), executive control network (ECN), default mode network (DMN), medial visual network (MVN), lateral visual network (LVN), cerebellar network (CBLN), salience network (SN), task positive network (TPN), basal ganglia network (BGN), frontal cortical network (FCN), anterior insula network (AIN). In this and the following figures, the 3D scans are projected onto three representative sections chosen on the sagittal (left), coronal (middle) and axial (right) planes.

The 15 RSNs were (Figure 1): auditory network (*AN*), sensory motor network (*SMN*), medial visual network (*MVN*), lateral visual network (*LVN*), task positive network (*TPN*), executive control network (*ECN*), right and left ventral attention networks (*VAN*), default mode network (*DMN*), salience network (*SN*), cerebellar network (*CBLN*), basal ganglia network (*BGN*), frontal cortical network (*FCN*), anterior insula network (*AIN*), language network (*LN*).

The *AN* is responsible for auditory processing and is located in the bilateral superior temporal gyrus, involving the primary and secondary auditory cortices. The *SMN* is responsible for sensory-motor processing and includes the primary somato-sensory, primary motor, premotor, and supplementary motor cortices as well as the cerebellum. The *LVN* and *MVN* are the networks responsible for visual processing, and are located in the lateral and medial parts of the occipital lobe, respectively, involving secondary and primary visual cortex, and also involve the cerebellum. The *TPN* is activated during task-oriented behaviors and includes bilateral dorsolateral and ventral prefrontal cortex, insula and primary sensory-motor areas. The *ECN* is responsible for executive processing, including perception, action selection, memory retrieval and emotional evaluation: it includes bilaterally the superior, middle and ventro-lateral prefrontal cortices, the anterior cingulate, and the paracingulate gyri. The *VAN* is the networks involved in the attentional processing: *VAN (left and right)* is largely lateralized in the temporal-parietal junction, in the ventral frontal cortex, in the insula and in the contralateral cerebellum. The *SN* mediates the function of other networks and includes bilaterally the anterior cingulate cortex, premotor, -supplementary motor areas, and anterior insula (Bonnelle et al., 2012). The *DMN*, which is active during episodic and autobiographical memory retrieval and shows decreased activity during cognitive tasks demanding attention to external stimuli (Raichle et al., 2001; Greicius et al., 2003, 2004), includes the bilateral inferior parietal cortex, the precuneus, anterior and posterior cingulate cortex, mesial-temporal structures including dorso-lateral prefrontal cortex, thalamus and cerebellum. The *BGN* is involved in emotional processing and includes the amygdala, striatum and lateral globus pallidus, mammillary bodies, hypothalamus, and the ventral tegmental area of midbrain. The *FCN* is involved in core mental functions at the basis of human social behaviors (Spreng et al., 2009) and includes the bilateral anterior cingulate cortex and boundary areas between prefrontal cortex (middle and inferior frontal gyri) and orbito-frontal cortex (Janes et al., 2012). The *AIN* is involved in cognitive control and orienting attention (Corbetta et al., 2002) and (Cole and Schneider, 2007) includes bilaterally the areas of insula, inferior frontal gyrus, anterior cingulate, the superior temporal gyrus, and cerebellum. The CBLN network is intrinsic to cerebellum and includes bilaterally large areas centered in crus I and crus II, cerebellar tonsil, the IV, V, VI, VIII, and IX lobes, dentate, declive, vermis, culmen, uvula, tuber, pyramis, medulla, and nodule. Finally, the LN involves the bilateral post cerebellum and inferior parietal lobule, the left inferior temporal gyrus and precuneus, as well as the inferior, middle, and superior frontal giri.

*LVN*, *MVN*, *AN*, *TPN*, and *SMN* are networks directly related to sensori-motor processing, while *DMN*, *SN*, *FCN*, *ECN, AIN*, *BGN*, *VAN*, and *LN*, are prominently associated with higher cognitive functions. The cerebellum showed components not only in the intrinsic *CBLN* but also in *SMN*, *MVN*, *LVN, DMN*, *AIN*, *VAN*, *LN* in line with the involvement of cerebellum both in sensory-motor and cognitive processing (Schmahmann et al., 1999; D'Angelo and Casali, 2012; Stoodley, 2012; Stoodley et al., 2012).

### Evaluation of group differences within the RSNs

**Figure 2:**
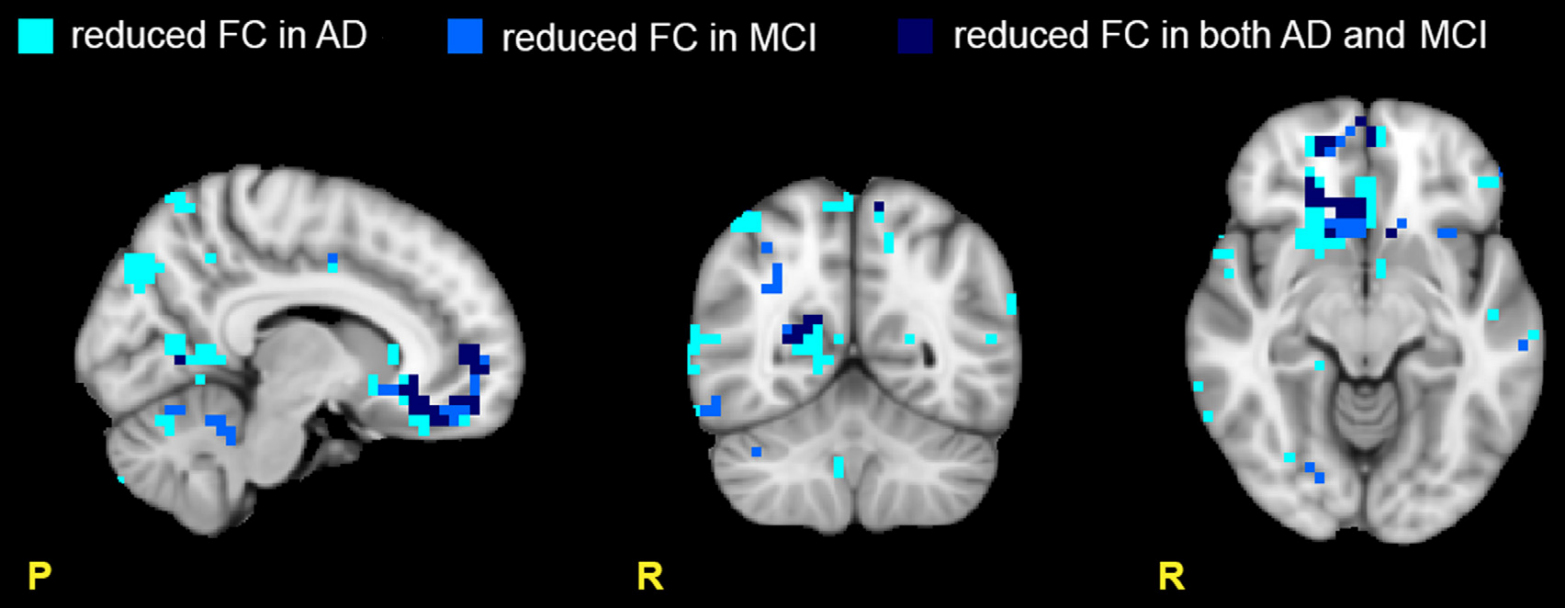
**Areas with reduced FC in the RSNs**. Images show areas with reduced FC (*p* ≤ 0.05, TFCE-corrected) in AD compared to HC (light blue), in MCI compared to HC (blue), and in both AD and MCI compared to HC (dark blue). Note that the areas of reduced FC are mainly located in the prefrontal (PreF) and precentral (PreC) areas. Moreover ADs present a larger number of areas with reduced FC than MCI suggesting a more extended functional corruption in AD than MCI.

**Figure 3:**
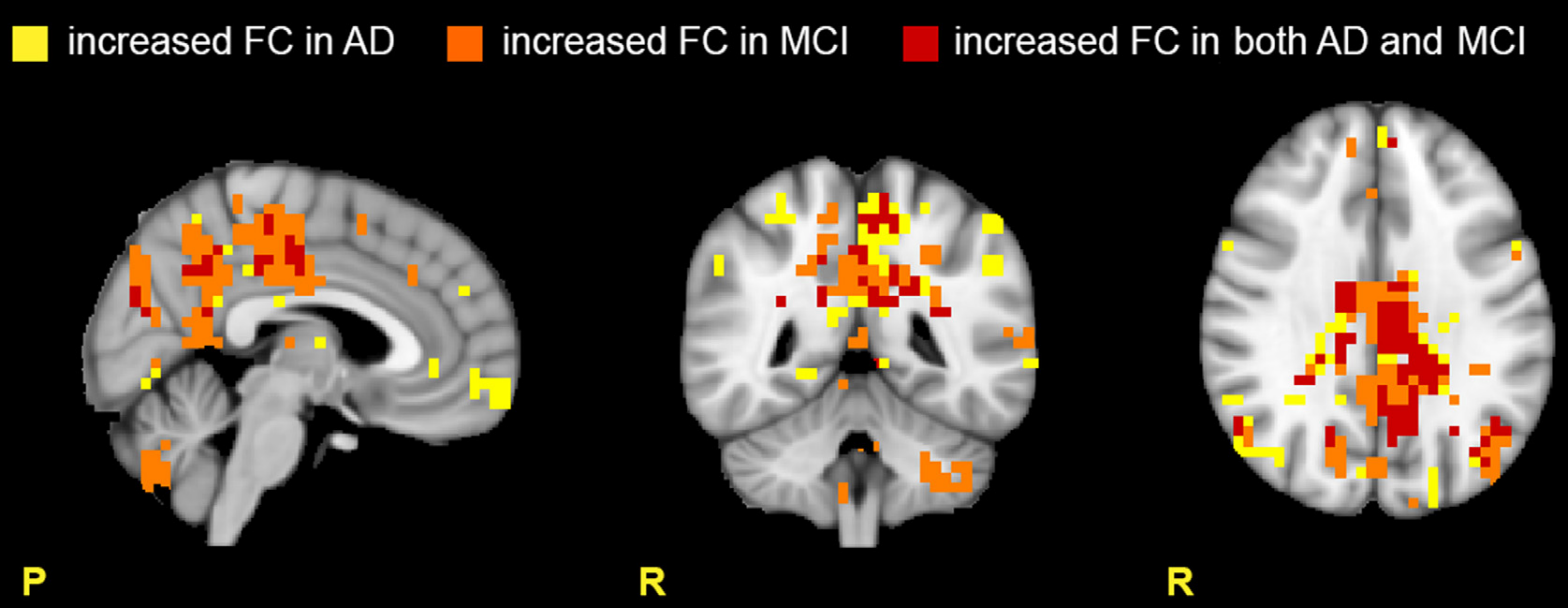
**Areas with increased FC in the RSNs**. Images show areas with increased FC (*p* ≤ 0.05, TFCE-corrected) in AD compared to HC (yellow), in MCI compared to HC (orange), and in both AD and MCI compared to HC (red). Note that the cerebellum presents large areas of increased FC in MCI, suggesting active compensatory mechanisms.

Our results are summarized in Table 3 and revealed, both in MCI and AD, a widespread FC alteration involving all the 15 RSNs. As shown in Figures 2, 3, depending on the RSN there was either a FC reduction or a FC increase, or both (in different sub-regions of the same RSN).

**Table 3:** **FC alterations within the 15 RSNs**.

		**AD < HC**	**MCI < HC**	**AD > HC**	**MCI > HC**
**RSNs**	**Total number of voxels (*N*_*RSN*_)**	***N*_*tstatFC*_ (%)**	***N*_*tstatFC*_ (%)**	***N*_*tstatFC*_ (%)**	***N*_*tstatFC*_ (%)**
R-VAN	3305	80 (2.42)	92 (2.78)	28 (0.85)	7 (0.21)
L-VAN	1751	45 (2.57)	5 (0.29)	45 (2.57)	6 (0.34)
BGN	926	30 (3.24)	0 (0.00)	140 (15.12)^**^	94 (10.15)^**^
LN	1741	13 (0.75)	24 (1.38)	67 (3.85)	21 (1.21)
AIN	808	3 (0.37)	3 (0.37)	103 (12.75)^**^	91 (11.26)^**^
SMN	1223	30 (2.45)	12 (0.98)	19 (1.55)	10 (0.82)
DMN	1819	441 (24.24)^*^	55 (3.02)	340 (32.08)^**^	410 (38.68)^**^
SN	1736	81 (4.67)	22 (1.27)	28 (1.61)	57 (3.28)
ECN	1408	44 (3.13)	0 (0.00)	0 (0.00)	5 (0.36)
FCN	1173	396 (33.76)^*^	197 (16.79)^*^	0 (0.00)	0 (0.00)
MVN	1980	2 (0.10)	0 (0.00)	87 (4.39)	23 (1.16)
AN	1368	19 (1.39)	0 (0.00)	87 (6.36)	8 (0.58)
LVN	1809	0 (0.00)	7 (0.39)	1 (0.06)	1 (0.06)
TPN	2562	3 (0.12)	45 (1.76)	158 (6.17)	2 (0.08)
CBLN	1655	47 (2.84)	1 (0.06)	0 (0.00)	153 (9.24)^**^

* *Largest FC decreases (prefrontal areas: inferior, middle, and superior frontal giri)*.

** *Largest FC increases (cuneus, precuneus, cerebellum)*.

#### Reduced FC in multiple RSNs

Several RSNs presented a FC reduction both in MCI (for a total of 474 voxels affected) and in AD (for a total of 1244 voxels affected), but the areas of reduced FC were less extended in MCI than AD. 17.76% of the total area of reduced FC in AD was also reduced in MCI, spreading over several networks. Although broad cortical regions were involved, the largest FC reductions were localized in the prefrontal cortex (i.e., PreF, including inferior, middle and superior frontal gyri) and in the precentral cortex (PreC), therefore involving primarily the FCN, both in AD (25.06 % of the total extension of the FCN) and MCI (13.90 % of the total extension of the FCN), (Figure 2).

#### Increased FC in multiple networks

Several RSNs presented an FC increase both in AD (for a total of 1711 voxels affected) and MCI (for a total of 1627 voxels affected). The 37.22% (627 voxels) of the total area of increased FC in AD was also increased in MCI, spreading over several networks. An interesting observation is that the RSNs showing a FC increase in patients compared to HCs the most included part of the cerebellum (C), which also showed voxels of increased FC (Figure 3).

### Global network analysis

#### Ranking of gFC changes in MCI and AD

**Figure 4:**
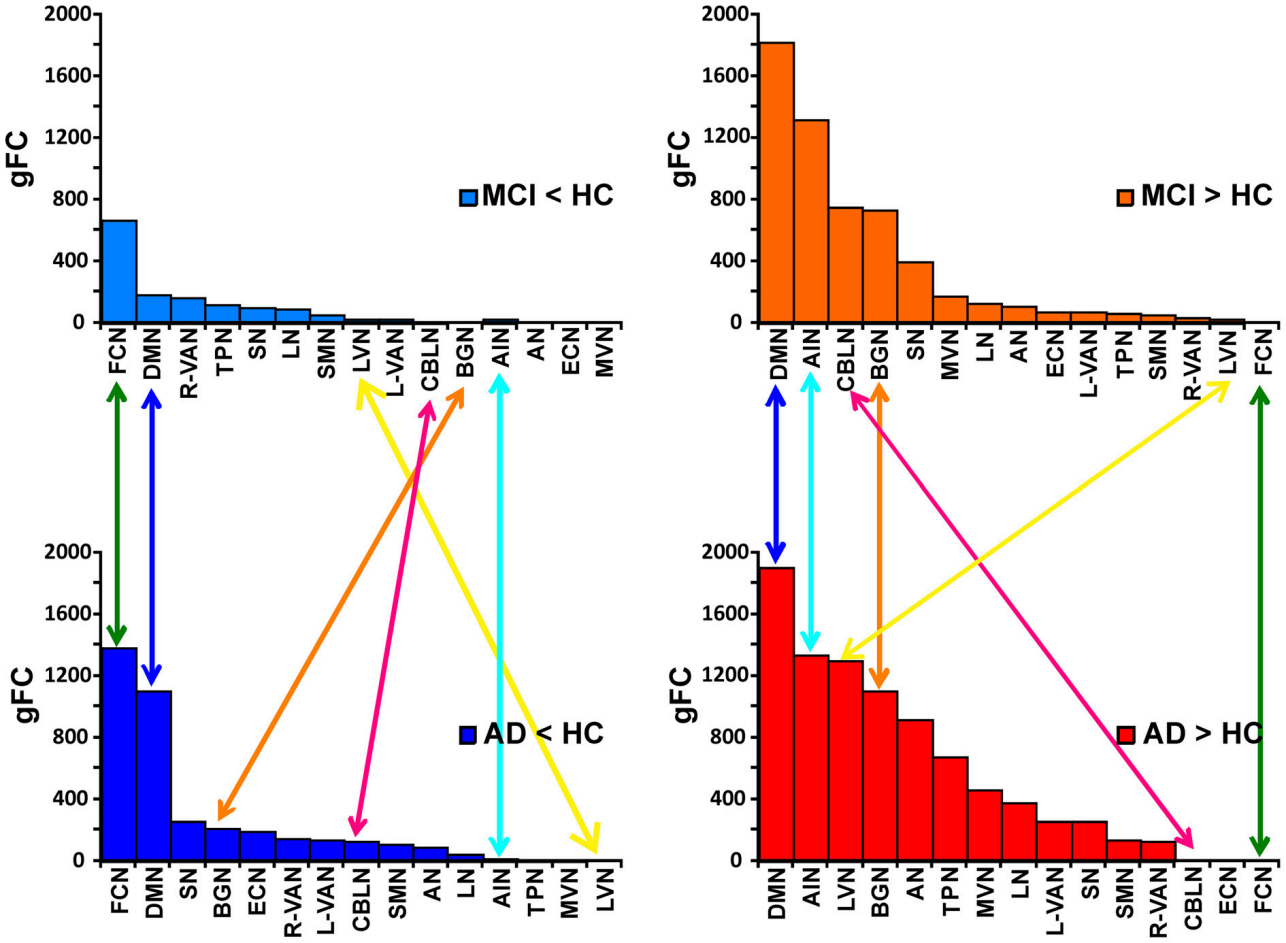
**Ranking of *gFC* changes in MCI and AD**. In order to better identify the patterns of *gFC* changes within the networks, for each contrast (MCI < HC, MCI > HC on the top; AD < HC, AD > HC on the bottom of the picture) we ranked the RSN alterations in terms of their decreasing *gFC*. We identified 6 different prototypical patterns: (1) FCN (green arrows) showed the largest *gFC* reduction, both in AD and MCI, and no increase in any conditions; (2) AIN (turquoise arrows) showed no *gFC* reduction, either in AD or MCI, but it showed a large increase in both conditions; (3) DMN (blue arrows) showed the second largest *gFC* reduction, both in AD and MCI, but at the same time it showed also the largest increase in both conditions; (4) BGN (orange arrows) shows a reduction in AD but not in MCI, while it also shows an increase in both cases; (5) CBLN (magenta arrows) shows just a minor decrease in AD but a remarkable increase in MCI only; (6) LVN (yellow arrow) shows *gFC* increase in both AD and MCI, with stronger effect in AD. At the same time it showed a small *gFC* reduction in MCI, but not in AD.

In order to better identify the patterns of alterations within the RSNs, we assessed the behavior of the *gFC* index for each of the four *contrasts*. Results, which are summarized in Figure 4, lead to the identification of 6 RSN with prototypical patterns:

1. The FCN showed the largest *gFC* reduction, both in MCI and AD, and no increase at all.
2. The AIN, on the contrary, showed no *gFC* reduction at all, but a large increase in both MCI and AD.
3. The DMN showed the second largest *gFC* reduction, twice as large in AD than in MCI, but at the same time it showed also the largest increase of *gFC* in both AD and MCI.
4. The BGN showed a reduction of *gFC* only in AD but not in MCI, while it also showed an increase of *gFC* in both cases.
5. The LVN showed mainly an increase of *gFC* in AD.
6. The CBLN showed just a minor reduction of *gFC* in AD but a remarkable increase of *gFC* in MCI only.

The other RSNs showed patterns similar to or between the prototypes identified here, with more modest alterations of *gFC* than these six networks. In particular, it is worth noticing that the SN has a pattern similar to the CBLN, where a reduction of *gFC* was seen in AD but not so much in MCI, while an increased *gFC* was observed mainly in MCI. These alterations, though, are less than half in size than similar changes in *gFC* in the CBLN.

### Individual network analysis

#### Patterns of rgFC changes in RSNs of MCI and AD

**Figure 5:**
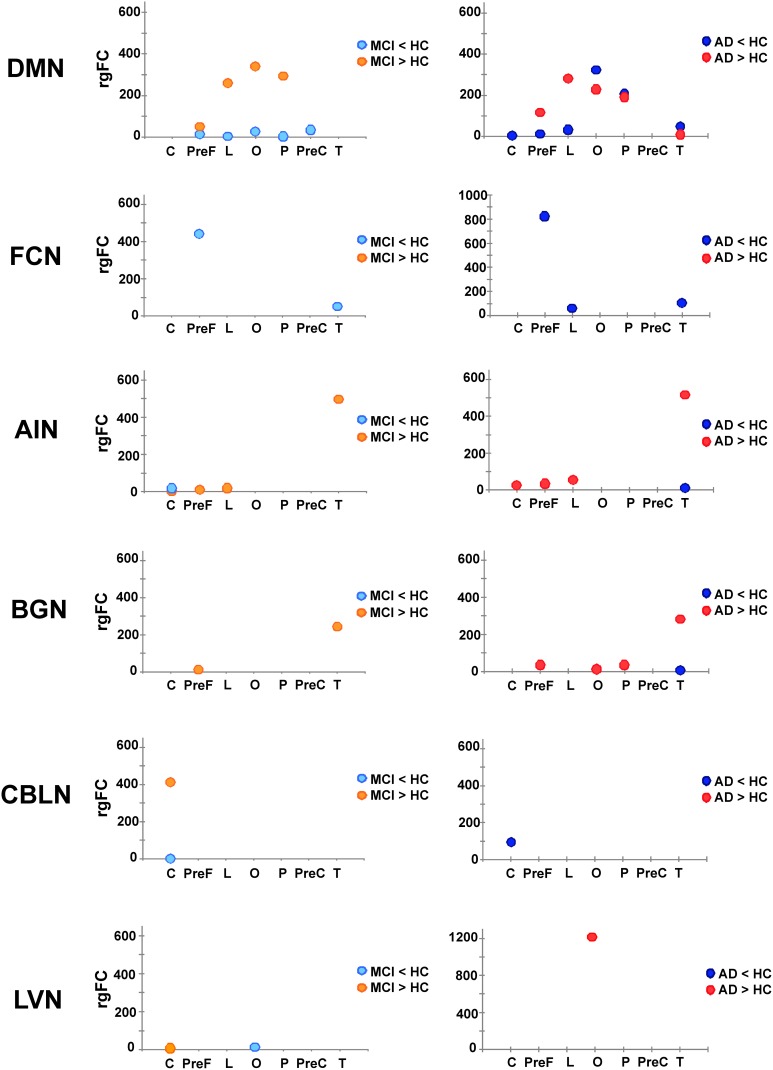
**Patterns of r*gFC* changes within specific RSNs**. Pictures show the value of r*gFC* across conditions (MCI < HC and MCI > HC on the left side, AD < HC and AD > HC on the right one) for each sub-area of the brain: cerebellum (C), prefrontal (PreF), limbic (L), occipital (O), parietal (P) precentral (PreC) and temporal (T) for the networks that showed specific prototypical patterns: DMN, FCN, AIN, BGN, CBLN, and LVN.

Once the 6 RSNs were identified through the *gFC* ranking, these were investigated in terms of the involvement of specific subcortical regions with the r*gFC* index defined above in the methods. Figure 5 gives a visual assessment of the involvement of each sub-area of the cortex in terms of decreased or increased r*gFC*.

In particular:

1. In the FCN, in both AD and MCI, a decrease of the r*gFC* was mainly in the prefrontal cortex (PreF), with a small reduction also in the temporal cortex (T). This effect was stronger in AD than MCI, i.e., r*gFC* changes in AD are twice the size of MCI changes. In AD, there was a further area of r*gFC* reduction corresponding to the limbic cortex (L). No r*gFC* increase was detected in any area of this network.
2. In the AIN, very small areas of r*gFC* decrease were found in the temporal cortex (T) in AD as well as in the cerebellar cortex (C) in MCI. On the other hand, the AIN was indeed the top-scoring network, after the DMN, when ranking them by their overall *gFC* increase (Figure 4). The regional assessment showed that the r*gFC* increase occurred predominantly in the temporal cortex (T) in both AD and MCI with stronger effects in AD than MCI, but also in the cerebellum (C), prefrontal cortex (PreF) and limbic cortex (L) in both AD and in MCI.
3. In the DMN, alterations both in terms of decreased and increased r*gFC* values affected several cortical areas. We found that r*gFC* decreased mainly in the parietal (P) and occipital cortices (O), including cuneus and precuneus areas, with stronger alterations in AD than MCI. Smaller r*gFC* reductions were found in the limbic cortex (L) and in the prefrontal cortex (PreF) in both AD and MCI, where it also extended to precentral cortex (PreC). In AD, there was a further area of r*gFC* reduction in the cerebellum (C) involving the left VI-V lobes and culmen. On the other hand, the DMN showed the areas of major r*gFC* increase in the limbic (L) and in the parieto-occipital cortices (P and O) in both AD and MCI, with stronger alterations in MCI than AD. Other areas of increased r*gFC* were found in the prefrontal cortex (PreF) in both AD and MCI, with stronger effects in AD than MCI, and in the temporal cortex (T) of the AD group.
4. In the BGN, the r*gFC* decreased only in the temporal cortex (T) in AD. The r*gFC* increased mainly in the temporal cortex (T) and in the prefrontal cortex (PreF) in both AD and MCI. These effects were stronger in AD than MCI. Further areas of increased r*gFC* were observed in AD in the parietal (P) and occipital (O) cortices.
5. In the LVN, a small area of r*gFC* decrease was observed in the occipital cortex (O) in MCI. No r*gFC* reductions were observed instead in AD. On the other hand, the r*gFC* increase was shown mainly in the occipital cortex (O) of AD. In MCI, there was only a small area of r*gFC* increase in the cerebellum (C).
6. In the CBLN, all changes happened at cerebellar level (C) because this network does not involve any other region of the cortex. Here, the r*gFC* decrease was greater in AD than MCI, while the r*gFC* increase was strong (third RSN in the ranking by *gFC* increase) and present only in MCI.

Although the meaning of r*gFC* increase or decrease in physiological terms is not fully clarified (see Discussion), there was a clear relationship between r*gFC* changes and the worse phase of the pathology.

#### Nodes of alterations

If we consider all the RSNs obtained with the dual regression analysis it is evident that there are several areas that functionally contribute to more than one network and as such can be considered as nodes or hubs. In particular, the sub-area analysis of r*gFC* alterations has highlighted three possible nodes, which are involved in alterations of several networks. These are: the prefrontal cortex (PreF: 2.22% of prefrontal alteration in AD and 1.09% in MCI), the mesial-temporal cortex (T: 2.33% of mesial temporal alteration in AD and 1.05% in MCI) and the parietal cortex (P: 1.76% of parietal cortex alterations in AD and 1.11% in MCI).

**Figure 6:**
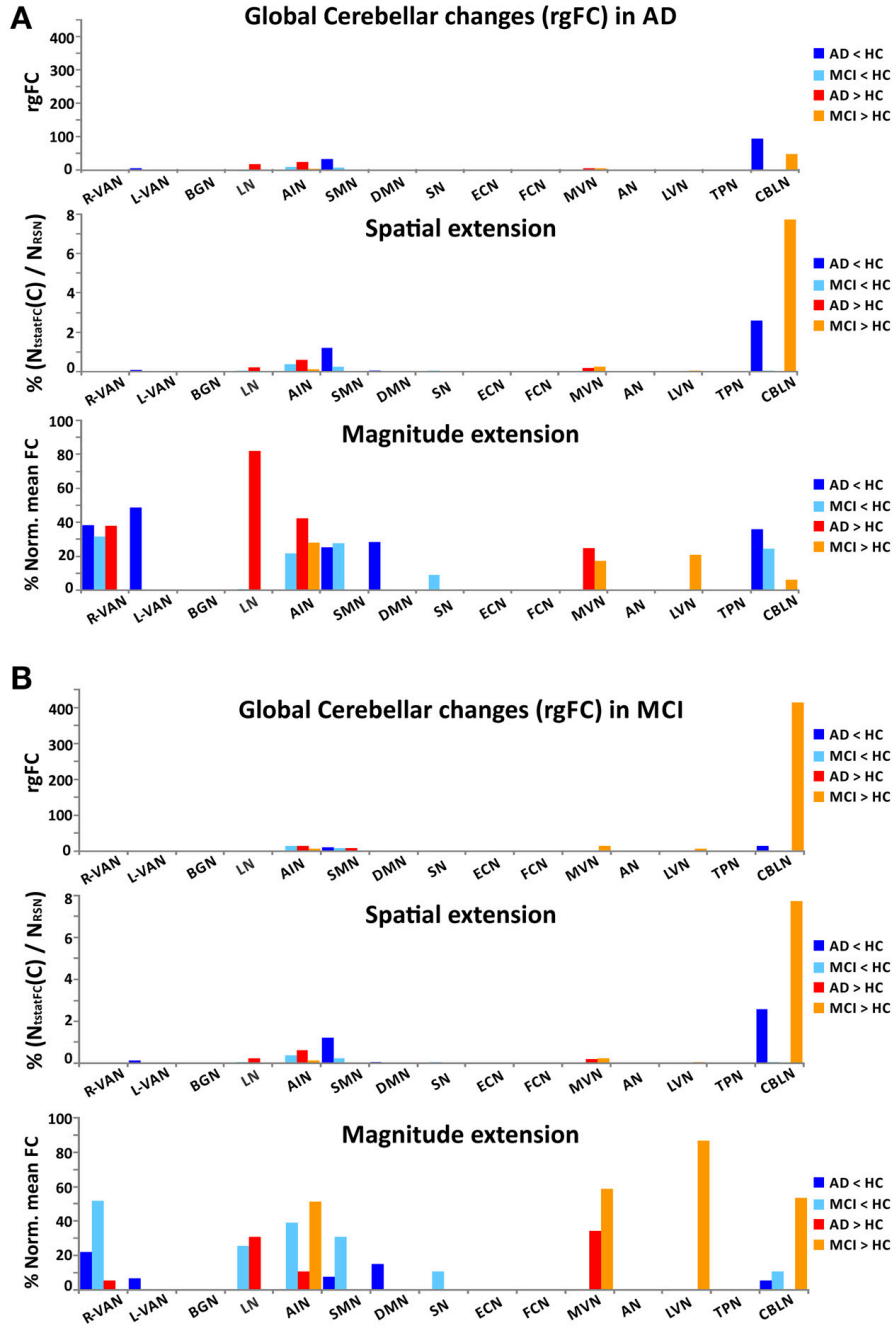
**Global cerebellar alterations in AD and MCI**. For each RSNs and for each contrast (AD < HC, AD > HC, MCI < HC, and MCI > HC), the histograms show the global changes occurring in the cerebellum C(r*gFC*) in AD **(A)** and in MCI **(B)**. For each RSN, the set of histograms shows: (1) the global changes of the r*gFC* index in the cerebellum (C), (2) the contribution to the r*gFC* in the cerebellum given by the *spatial extension* of the clusters of functional alteration (*N*_*tstatFC*_/*N*_*RSN*_), (3) the contribution of the *magnitude extension* of the cerebellar functional alteration [*meanFC*(C)_*P*_with p = MCI or AD, normalized for the to the *meanFC*(C)_*HC*_].

Although the cerebellum (C) doesn't emerge as one of the principal nodes of r*gFC* alteration (C: 0.41% of cerebellar alteration in AD and 0.79 % in MCI), it is often involved both in MCI and AD showing altered r*gFC* values across several RSNs (Figure 6). There were, in fact, 10 RSNs presenting areas of r*gFC* changes in the cerebellum (C): the CBLN, the DMN, the AIN, the SN, the MVN, the LN, the VANs, the SMN, and the LVN. MCI showed the major areas of r*gFC* increase in DMN, AIN and CBLN with the most prominent r*gFC* increase in the DMN. AD showed areas of r*gFC* decrease particularly in DMN, SN, CBLN, VANs, and SMN but also a large area of r*gFC* increase in the DMN.

### VBM structural changes

**Figure 7:**
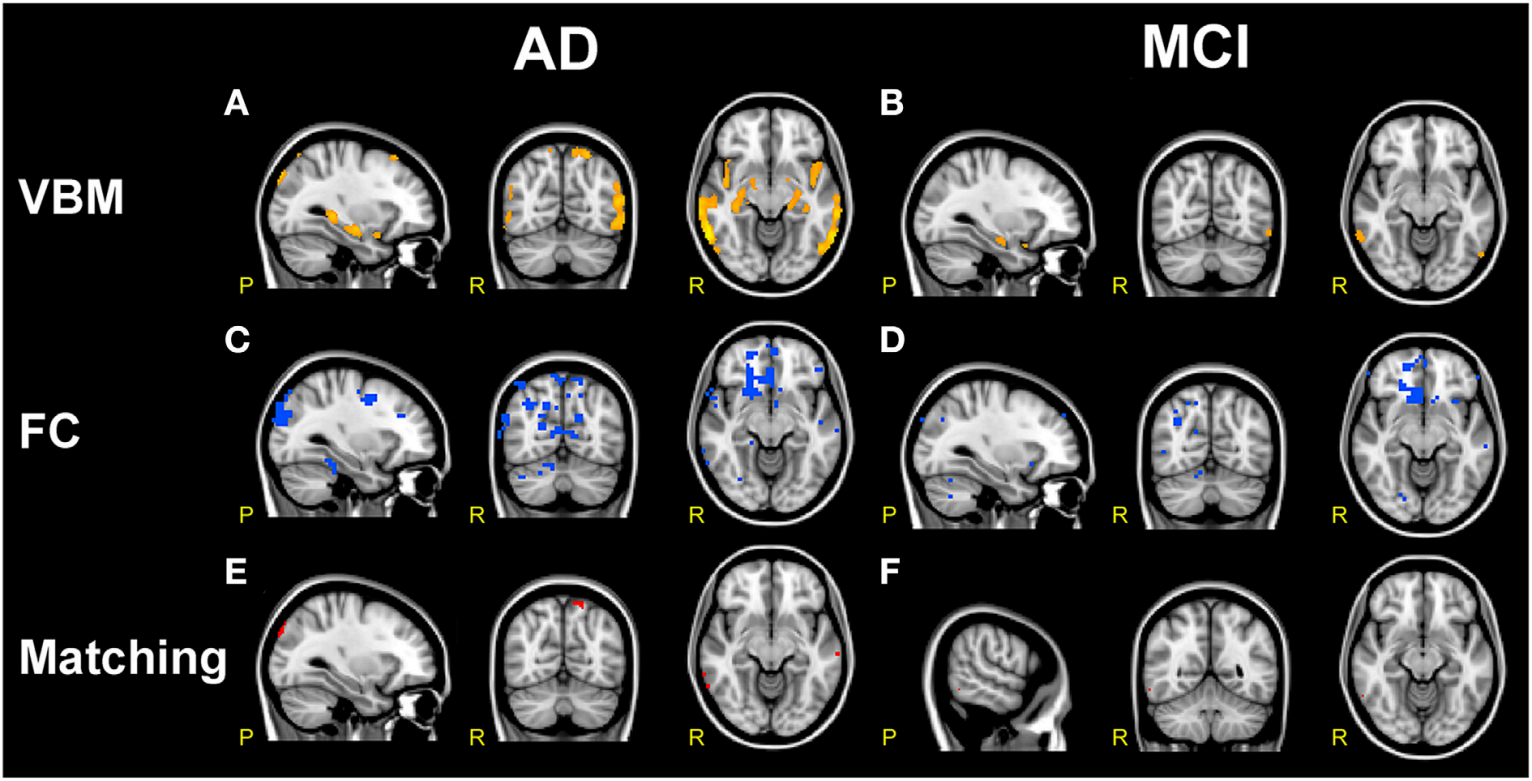
**Comparison of structural and functional changes in AD and MCI. (A,B)** *VBM analysis*. AD subjects compared to HC **(A)** show large areas of GM atrophy (orange) in bilateral mesial-temporal network. MCI subjects compared to HC **(B)** show sparse areas of GM atrophy (orange) in left and right hippocampus, left amygdala, parahippocampal gyrus, precuneus and superior temporal gyrus. AD show more extended areas of atrophy than MCI. **(C,D)** *FC analysis*. Both AD and MCI compared to HC show large clusters of FC reductions (blue) mainly localized in the prefrontal areas involving VAN, DMN, DAN, FCN, SN, and SMN, with more extended areas of FC reductions in AD than MCI. **(E,F)** *VBM/FC matching*. Matching VBM and FC maps of FC reduction for AD compared to HC and MCI compared to HC. The overlapping between atrophy and FC changes (red clusters) is very limited.

VBM analysis in AD subjects (compared to HC) showed large areas of GM atrophy mainly in the left and right hippocampi, left amygdala, parahippocampal gyrus, uncus, inferior, middle, superior, and transverse temporal gyri (Figure 7A), as well as in smaller areas in the right amygdala and precuneus. VBM analysis in MCI subjects (compared to HC) showed areas of GM atrophy mainly in left and right hippocampi and in the left amygdala, the parahippocampal gyrus, the precuneus, and the superior temporal gyrus (Figure 7B). As a whole, GM atrophy was more extended in AD (68,736 mm^3^) than MCI (8,184 mm^3^) with a ratio approximately of 8:1.

It should be noted that GM atrophy was significantly detected in a large area of the bilateral mesial-temporal cortex (*p* < 0.001, FDR-corrected) and that, although both AD and MCI were atrophic in the superior temporal gyrus and in the mesial-temporal areas (left and right hippocampi, amygdala, and parahippocampal gyrus), AD presented larger clusters of atrophy than MCI. In particular AD subjects compared to MCI were more atrophic in the superior and middle temporal gyri. These results substantially confirmed previous reports (Ries et al., 2008; Ferreira et al., 2011) and revealed that areas of GM atrophy overlap at the 6.8% with the DMN, although only a small portion (1.65%) of this areas of GM atrophy were found co-localized with FC alterations.

In particular, the comparison of structural and functional changes in AD subjects (compared to HC) showed that GM atrophy was consistent with the FC decrease observed in the BA 20, 21, and 22 in the inferior, middle and superior temporal gyri (T), in the right parahippocampal gyrus, in the BA 10 in the prefrontal areas (PreF), in the cuneus and in the left precuneus (Figure 7E). Similarly, the comparison of structural and functional changes in MCI subjects (compared to HC) showed that GM atrophy is consistent with the FC decrease observed in the middle and inferior temporal gyrus (T), although it should be noted that the overlapping between atrophy and FC changes resulted very limited (see Figure 7F).

### Correlation between functional connectivity and the clinical and neuropsychological state

**Figure 8:**
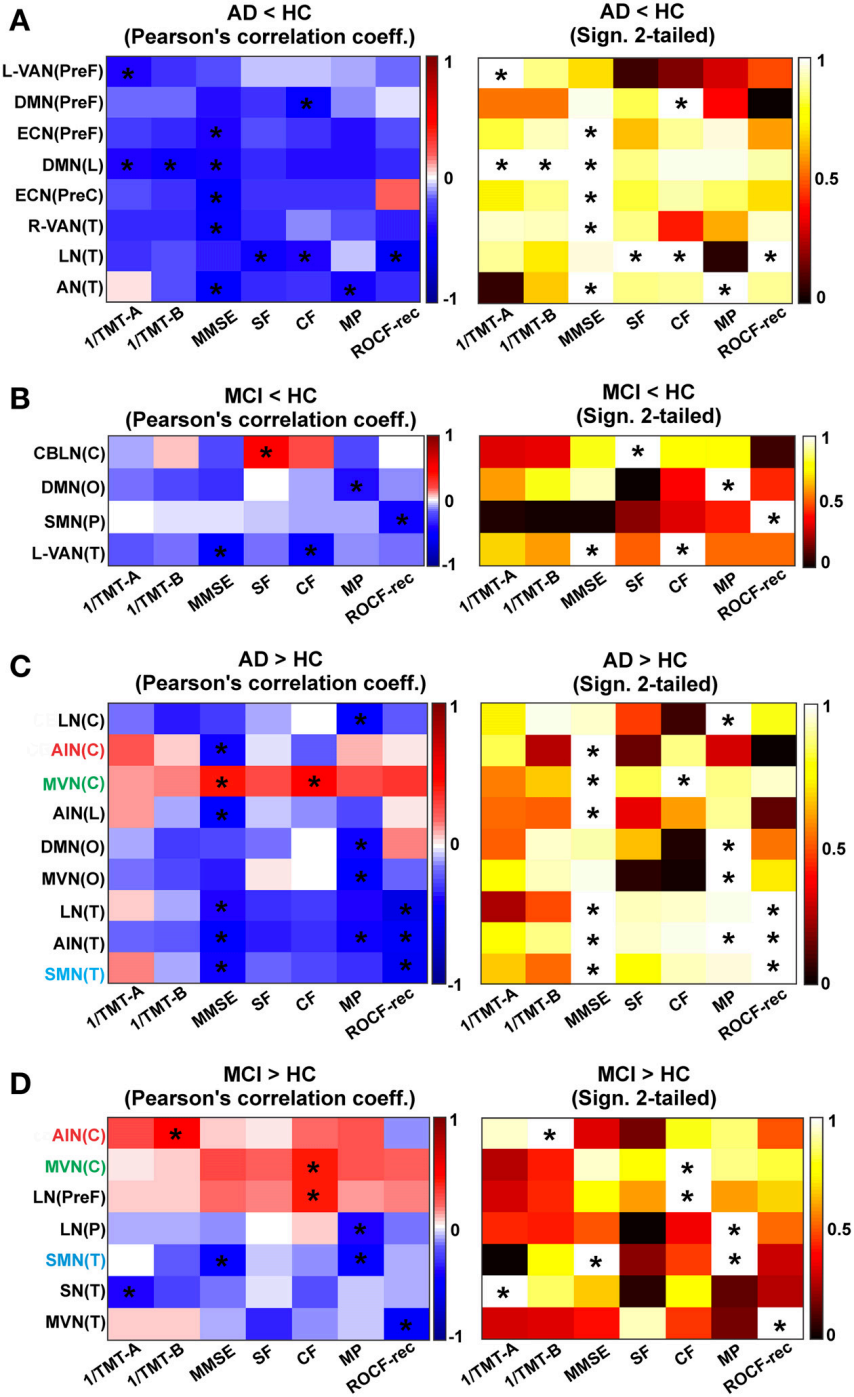
**Correlation between r*gFC* changes and clinical and neuropsychological tests**. Results are reported for each contrast **(A)** AD < HC, **(B)** MCI < HC, **(C)** AD > HC and **(D)** MCI > HC. Correlation matrixes (left) and the corresponding statistical significance of correlations (right) between clinical and neuropsychological tests (1/TMT-A, 1/TMT-B, MMSE, MP, CF, SF, ROCF-copy, and ROCF-rec) and r*gFC* areas: cerebellum (C), prefrontal (PreF), limbic (L), occipital (O), parietal (P), precentral (PreC), and temporal (T). Correlation and anti-correlation are reported on different color scales. Correlations that satisfied a statistical threshold of *p* < 0.01 are marked with a star (^*^). The statistical significance (matrices on the right) of each Pearson's correlation coefficient (matrices on the left, with the positive correlations in red scale and the negative ones on blue scale) is represented as 1–*p*-value.

In order determine whether a relationship existed between the complex changes in brain r*gFC* and the pathological state of the AD and MCI subjects, a cross-correlation matrix was generated (Figure 8).

#### Correlations in AD (compared to HC)

In AD (compared to HC) the MMSE, SF, CF, MP, TMT-A, TMT-B, and ROCF-rec indexes were found to correlate (*p* < 0.001) with the mean r*gFC* value in areas of reduced r*gFC* in the PreF, PreC, T and L areas of multiple networks (Figure 8A). The MMSE showed the major number of significant correlations with reduced r*gFC* over some of the most relevant brain areas for AD (PreF, PreC, T, and L). In detail, the MMSE inversely correlated (*p* < 0.001) with reduced r*gFC* in the DMN(PreF) and DMN(L), in the ECN(PreF) and ECN(PreC) and in the R-VAN(T), the AN(T) and the LN(T). Areas of decreased r*gFC* in the DMN(L) showed a strong inverse correlation with both 1/TMT-B and 1/TMT-A [which, also inversely correlated with reduced r*gFC* in the L-VAN(PreF)]. Both the SF and the CF inversely correlated with decreased r*gFC* in the LN(T), while the MP showed a strong inverse correlation with reduced r*gFC* of the AN(T). Finally, the ROCF-rec was found to inversely correlate with decreased r*gFC* in the LN(T) and positively correlate with the ECN(PreC).

Moreover in AD, the MMSE, MP, ROCF-rec and the CF showed a major number of correlations with increased r*gFC* over different RSN brain areas (Figure 8C). The MMSE inversely correlated with increased r*gFC* in the AIN(C), AIN(L) and AIN(T), in the LN(T) and SMN(T), and in the DMN(O). An inverse correlation was also observed between the CF, MP, and ROCF-rec and increased r*gFC* in the AIN(T). The ROCF-rec inversely correlated also with increased r*gFC* in the LN(T) and the SMN(T). Moreover, increased r*gFC* in the DMN(O) and the MVN(O) inversely correlated with the MP. Moreover the MP and the 1/TMT-B inversely correlated with increased r*gFC* in the LN(C). A positive correlation was observed instead between all neuropsychological tests and the MVN(C), with a particularly strong positive correlation between the MMSE and the CF indexes with an increased r*gFC* in the MVN(C).

#### Correlations in MCI (compared to HC)

In MCI, a reduced number of significant correlations, considering a *p* < 0.001, were observed between the neurological indexes (MMSE, CF, MP, ROCF-rec, and SF) and reduced r*gFC* areas over the brain compared to AD as shown by a reduced matrix size (Figure 8B compared to Figure 8A). In detail, in MCI areas of reduced r*gFC* in the L-VAN(T) showed an inverse correlation with the MMSE and the CF. Significant inverse correlations were observed between the MP and reduced r*gFC* in the DMN(O), as well as between the ROCF-rec and reduced r*gFC* in the SMN(P). A positive correlation was found instead between the SF and reduced r*gFC* in the CBLN (C).

Furthermore, in MCI the 1/TMT-A, 1/TMT-B, MMSE, CF, MP, and the ROCF-rec showed at least a significant correlation with increased r*gFC* over different RSN areas (Figure 8D). In particular, strong inverse correlations were observed between the ROCF-rec and increased r*gFC* in the MVN(T), as well as between the MMSE and the SMN(T). The 1/TMT-A also anti-correlated with increased r*gFC* in the SN(T), while the MP showed strong inverse correlation with increased r*gFC* in the SMN(T) and in the LN(P). The CF showed instead a positive correlation with increased r*gFC* in the MVN(C) and in the LN(PreF), as well as the 1/TMT-B was found to correlate positively with the AIN(C).

Comparing trends of correlations between mean r*gFC* in areas of increased r*gFC* and neuropsychological scores in AD and MCI, three are the areas that are emerging as being correlated in both groups of patients (Figures 8C,D): MVN(C), SMN(T), and AIN(C). The MVN(C) seems to present a similar trend in MCI and AD for all tests. The SMN(T) instead shows an inverse correlation between r*gFC* increase and MMSE in both groups, while AIN(C) shows a positive correlation between r*gFC* increase and CF scores in MCI and an inverse correlation between r*gFC* and CF scores in AD. Similarly there is a positive correlation between the r*gFC* increase and MMSE in MCI, which instead is an inverse correlation in AD.

### Correction for relative GM volume

All the clusters of FC reductions and FC increases, described so far, survived statistical thresholding (*p* < 0.05, TFCE-corrected) when correcting for GM atrophy.

## Discussion

A hallmark of AD and MCI in rs-fMRI studies is the FC reduction in the DMN, which was confirmed by our findings that showed a reduced FC connectivity in a large temporo-parietal (T and P) area, centered in the cuneus, precuneus and the posterior cingulate cortex, in AD and in MCI, although in MCI areas of FC disruption were found less extended (20.05% of the DMN was affected by FC reduction in AD, while only 3.17% in MCI).

In this paper we presented a global approach for a comprehensive assessment of alterations in FC of all RSNs and applied it to a pilot study of dementia patients. The introduction of a global parameter (the *gFC* index) was driven by the need to summarize both FC changes and voxels extension of such changes for each RSN and allowed us to (i) create a ranking of all RSNs, (ii) highlight which are the main RSNs undergoing significant patterns of alterations and (iii) evaluate the contribution of areas of the cerebral cortex and cerebellum to such changes in different pathological states.

The main observation in this pilot study is that multiple RSNs, rather than just DMN, are modified in AD and MCI patients presenting both patterns of decreased and increased FC changes which could be identified in six different prototypical RSNs. Furthermore, although a widespread FC change involved to some extent all the 15 RSN identified (namely *AN, ECN, VANs, LN, DMN, SMN, BGN, AIN, SN, FCN, LVN, MVN, TPN*, and *CBLN*) the sub-area analysis that we performed revealed the emergence of alterations in specific nodes (or hubs) including primarily the prefrontal cortex (PreF), the mesial-temporal cortex (T), and the parietal cortex (P). We also acknowledge the extensive involvement of the cerebellum (C) in several RSN disruptions. In addition to support a functional disconnection between prefrontal cortex (PreF) and hippocampus (T) (Wang et al., 2006; Zhang et al., 2009), our data revealed that both AD and MCI showed areas in which *gFC* was either reduced or increased, with a prevalent *gFC* decrease in AD but also an equally relevant *gFC* increase in both AD and MCI (see Figure 4). Noticeably the *gFC* increase affected also the DMN. Finally, these *gFC* changes correlated with the neuropsychological status of patients.

When dealing with FC increases, however measured (through the standard FC parameter or *gFC* or r*gFC*), it is tempting to interpret data as compensatory mechanisms taking place in MCI while network disruption prevailing in AD. This first justification is challenged in details below as it may be too simplistic in the presence of a complex pathophysiology such as in dementia. Interestingly, both the FC reductions and the FC increases survived statistical thresholding (*p* < 0.05, TFCE-corrected) when correcting for GM atrophy, indicating that the FC alterations we observed are likely to be independent from global GM atrophy. Also, FC changes overlapped only minimally with local GM reductions as shown by the VBM analysis.

While these results need confirming in larger clinical studies, they raise important issues such as: What is the nature of FC alterations in the absence of morphological alterations? Why do some areas show increased FC while others show decreased FC? What is the meaning of the cerebellum FC changes? Are there any specific correlations between the areas showing FC changes and the neuropsychological state of patients?

### Functional alteration in areas without significant cortical atrophy

As expected, GM atrophy was more extended in AD than MCI with a ratio approximately of 8:1. Both GM atrophy and FC reductions in AD and MCI affected in particular the prefrontal cortex (inferior, middle and superior gyri) and a large mesial-temporal-parieto-occipital (T-P-O) area (including hippocampus, parahippocampal gyrus, precuneus, cuneus, and amygdala). These observations are in line with others showing similar profiles in neuropathology, atrophy, metabolism and FC in the cortical surface of AD patients (Buckner et al., 2005; Ries et al., 2008; Ferreira et al., 2011). However, VBM analysis revealed that areas of reduced FC only partially overlapped with GM atrophy both in AD (333 voxels equals to the 3.78% of the GM atrophy area) and MCI (1 voxel equals to the 0.09% of the GM atrophy area).

Since there is a clear relationship between neuropathological signs (amyloid plaques and tau tangles) in the prefrontal cortex (PreF) and mesial-temporal (T) lobe and reduced cognitive performance in AD, the emergence of FC alterations in regions classically devoid of neuropathological alterations (e.g., several other cortical areas and the cerebellum) is puzzling. A possible explanation is that primary damage causes a subsequent derangement of functional relationships between multiple areas, a fact that should not be surprising as we are dealing with large-scale networks in which different areas are functionally interconnected. The prefrontal cortex and mesial-temporal lobe are hubs taking part in multiple functional networks, and their alteration might reverberate into widespread changes. Whatever the sign and mechanism of FC changes, a generalized alteration in RSNs is akin with the proposal that central neurodegenerative diseases, including AD eventually drive into a dysfunctional state large-scale networks extending beyond the initial core of neurodegeneration (Seeley et al., 2009).

### Seeking for patterns of FC change in RSNs of MCI and AD

Once moving beyond the concept that AD and MCI are characterized mainly by DMN changes, and once realizing that almost all the RSNs show alterations consisting of either an FC increase, decrease or both, the search for meaningful alteration patterns becomes complicated by the intersection of RSNs in multiple nodes. Moreover, the presence of FC changes that can differ in amplitude as well as spatial extent can complicate interpretation. We have assumed that both the absolute change of FC value and the extension of the change have a major contribution into network disruption. Within this framework we have introduced the generalized FC index (*gFC*), which has revealed general patterns of FC alterations summarized as follows: (i) the *gFC* decrease was about twice as large in AD then MCI, (ii) the *gFC* increase was about four times larger than the *gFC* decrease, (iv) the *gFC* increase was extensive both in MCI and AD.

Understanding how these FC changes could be generated is challenging. An hypothesis could be that changes start from the DMN and FCN, in which the FC decrease may reflect primary structural alterations already present in MCI and persisting in AD. Passing through the nodes of the DMN and FCN, the functional alterations may reverberate into other networks. Since there is a critical worsening of symptoms in AD compared to MCI, an FC decrease may correlate with reduced functionality. Given the extent of *gFC* increase in AD, as well as MCI, the interpretation of such an increase requires thoughts. On one hand, functional compensation may exploit the pre-existing neural reserve through plasticity and it would be easy to justify as a mechanism particularly active in MCI. Then, either due to hypo-activation or to a more extended micro-structural damage, more and more networks could run into a hypo-functional state reflecting the condition of AD with a larger decrease of *gFC*. This may be true for the CBLN, showing a net switch from increased *gFC* to decreased *gFC* when considering MCI or AD, and for the BGN, in which *gFC* reduction appears just in AD patients. On the other hand though, some RSN like the AIN show an increase *gFC* in both MCI and AD. This could be interpreted as a compensatory mechanism typical of MCI, but still surviving even in patients with advanced pathology such as AD. Although this hypothesis is attractive for its simplicity, the fact that *gFC* increase was even greater in AD than MCI suggests the possibility of a more complex scenario, in which functional degeneration and compensation may evolve dynamically presenting different patterns in different stages of the disease (Zhang et al., 2009; Bai et al., 2011a). An alternative interpretation of increased *gFC* in both MCI and AD is that it represents, at least to some extent, hyper-synchrony and phase locking, thereby reducing mutual information transfer through network nodes (Borst and Theunissen, 1999). Consistent with this hypothesis is the finding of diffused increase of spectral power in the EEG delta band of AD patients, which is also index of disease progression (Babiloni et al., 2004, 2014). In this case, the hypothesis of a compensatory mechanism may be erroneous. Rather, increased *gFC* may be an unsuccessful attempt of compensation, therefore may be interpreted itself as a disease expression.

To further evaluate these hypotheses, multidisciplinary approaches might be used; in particular positron emission tomography (PET) imaging for measures of glucose metabolism and combined electrophysiological and fMRI experiments should be devised. It would also be of interest to evaluate whether these FC changes are accompanied by corresponding structural changes in the tracts connecting cortical areas and the cerebellum.

### Involvement of the cerebellum in MCI and AD

The functional involvement of the cerebellum in AD is in line with the observations that MCI patients exhibit altered cerebello-thalamo-cortical activations during Stroop tests (Kaufmann et al., 2008), abnormal thalamo-cortico-cerebellar connections (Teipel et al., 2008) and significantly lower FC in a network involving hippocampus, prefrontal lobe, temporal lobe and parietal lobe (Bai et al., 2009). The cerebellum was also reported to undergo degenerative changes in AD, showing atrophy of the posterior cerebellar region associated with impaired cognitive performance. Therefore, our result supports the hypothesis that the cerebellum is involved in different forms of cognitive impairment including MCI and AD (Thomann et al., 2008).

Since the cerebellum is typically devoid of amyloid plaques (Serrano-Pozo et al., 2011; Ni et al., 2013), the RSN changes involving the cerebellum may be explained, at least in part, by abnormal recruitment of neurons caused by a primary cerebro-cortical change. Since in the cerebellum, and in particular the CBLN, a *gFC* increase prevails in MCI while a *gFC* reduction prevails in AD, the former may reflect a reaction to the altered relationship with the cerebral cortex, while the latter may be a direct consequence of functional disruption. It is tempting to speculate that increased cerebellar *gFC* takes part in a homeostatic mechanism that aims at limiting the progression of cognitive decline from MCI to AD, which can only be properly tested in longitudinal studies.

### Correlation of functional connectivity changes with the neuropsychological state

In line with MCI and AD clinical definitions, the major alterations observed at the neuropsychological level concerned attention and memory. These two functions are elaborated in extended brain networks involving the prefrontal cortex (PreF), mesial-temporal lobe (T) and cerebellum (C), which all showed altered activity in *DMN*, *FCN*, *VAN*, *LN*, *SMN*, *AIN*, *MVN*, *LVN, CBLN*. Among the tests, MMSE and MP and CF were those that showed the most significant correlations with r*gFC* changes. The MMSE showed the major number of significant correlations with most brain regions (PreF, PreC, T, L, O and C in AD; T and C in MCI). Moreover, the MMSE is also the only index to show more than one significant correlation (*p* < 0.001) with both r*gFC* increase and r*gFC* decrease in the cerebellum, which is known to be strongly interconnected with both motor and non-motor (associative) areas, including PreF and T cortices (Houk and Wise, 1995; Schmahmann et al., 1999; Habas et al., 2009; Krienen and Buckner, 2009; Stoodley and Schmahmann, 2010; Stoodley, 2012; Stoodley et al., 2012; Keren-Happuch et al., 2014). The cerebellum is important for operations of timing, prediction and learning and for integrating them into processes of novelty/error detection, working memory and mental manipulation (D'Angelo and Casali, 2012). This allows the cerebellum to take part not just to motor control but also to attention switching, language processing, imagery, decision making and reasoning, consistent with a potential involvement in the pathophysiology of MCI and AD.

The fact that both r*gFC* increase and r*gFC* reduction correlate with MMSE straighten the hypothesis that both r*gFC* modification are expression of disease, possibly in different diseases phases. In each cerebral area, the initial dysfunction may correlate with an attempt of compensation and a r*gFC* increase. Later in the disease progression, in the same area, also affected by atrophy, the r*gFC* decrease may predominate. It is indeed interesting that the correlations between MMSE and r*gFC* increase are mainly inverse in AD while some areas, including cerebellar regions [AIN(C) and MVN(C)] are showing a positive trend in MCI.

Comparing the matrices in Figure 8C and d at a glance it is easy to see that the matrix referring to correlations in AD is much more “blue” (inverse correlation) than the matrix for the MCI where almost half of the squares are “red” (positive correlation) and involve cerebellar areas. This could be an indication supporting the interpretation that an increased r*gFC* could be due to compensatory mechanisms in MCI but to phase locking of signal transmission within a network in AD. Interestingly, for example, there is a positive correlation between the r*gFC* increase in the AIN(C) and the CF score in MCI, while an inverse correlation in AD.

### Possible limitations of the present study

The results reported in the present investigation have to be confronted with some technical and methodological limitations. First of all, the relatively small sample size might have limited the statistical power to display changes in regional FC in AD and MCI groups. Moreover, the MCI population was a mixture of amnestic and non-amnestic subjects. Although we performed a strict quality control on our images, we recognize that our MCI population represents a late stage of this phase of the disease. Future studies should assess the RSNs changes in cohorts of prodromal dementia patients (Minati et al., 2014), performing also longitudinal studies to follow changes in RSNs of patients who convert or do not convert to AD. Further studies will also be needed to recruit a larger sample of patients, to subdivide amnestic from non-amnestic MCI subjects and to follow-up their clinical and MRI evolution in a longitudinal study. The MRI protocol was performed on a 1.5T clinical scanner with clinical protocols, rather than sequence parameters optimized for a research study. Current state of the art scanners (e.g., 3T) would allow better acquisition protocols, for example acquiring smaller isotropic voxels, in similar scan times. Moreover, it remains to be seen whether the FC findings in MCI are related to their possible progression to AD, providing a possible biomarker for cognitive dysfunction in AD-related illness evolution. Further investigations are also needed, including possible electrophysiological measurements, to help assessing the theory of compensatory mechanisms or reduced mutual information to explain increased FC in both MCI and AD.

Recent studies of metabolic energy demand of the resting human brain suggest that glutamatergic function in the resting awake human brain is supported by uniformly high oxidative energy (Hyder and Rothman, 2012; Hyder et al., 2013). Moreover, Tomasi and colleagues (Tomasi et al., 2013) showed that BOLD signal fluctuations with larger amplitudes are localized in brain regions characterized by higher metabolism. They also suggest that the higher energy demands of brain communication that hinges upon higher connectivity could render brain hubs more vulnerable to deficits in energy delivery or utilization and help explain their sensitivity to neurodegenerative conditions, such as AD (Tomasi et al., 2013). Other studies suggested, indeed, that there are organized patterns of brain metabolism, and the associated metabolic activity might reflect the relevant brain functions between the brain regions involved (Wu et al., 2009; Di et al., 2012). The above reported evidence suggests that rs-fMRI on AD and MCI should be associated to FDG-PET acquisitions in order to compare and discuss FC alterations with regard to metabolic deficiencies. Therefore, in future it could be useful to adopt multi-modal approaches combining measures of FC with indexes of fiber integrity and quantitative measures of glucose metabolism.

## Conclusions

We have successfully developed a strategy to assess multiple RSNs in terms of their FC changes (both in magnitude and spatial extension) as well as to evaluate contributions of different cortical regions to FC alterations. The picture that emerges from these rs-fMRI results suggests that MCI and AD FC changes occur in hubs laying at the intersection of multiple RSNs (all the 15 RSNs identified in the study), rather than just in the DMN, and encompass both decreased and increased FC. These hubs are mostly located in the prefrontal and parieto-temporal cortices. The cerebellum is also involved in several changes, which might suggest that it participates as well as various cortical areas to compensatory processes, although the hypothesis of reduced mutual information cannot be discarded, especially in the advanced stage of the disease such as in AD. Longitudinal studies on large patients groups are needed to investigate physiological meanings of changes and the correlation between RSN changes and disease development. The pilot study presented here, though, thoroughly demonstrated that the assessment of alterations in FC of multiple extended large-scale networks of the cerebral cortex and cerebellum using rs-fMRI deserves further attention and may provide new cues to examine the pathophysiology of neurodegenerative diseases and to determine the progression of MCI into AD.

### Conflict of interest statement

The authors declare that the research was conducted in the absence of any commercial or financial relationships that could be construed as a potential conflict of interest.

## Abbreviations

AD: Alzheimer disease
AIN: anterior insula network
AN: auditory network
BA: Brodmann area
BGN: basal ganglia network
CBLN: cerebellar network
LN: language network
DMN: default mode network
ECN: executive control network
HC: healthy controls
ICA: independent component analysis
LVN: lateral visual network
MCI: mild cognitive impairment
FCN: frontal cortical network
MVN: medial visual network
rs-fMRI: resting state functional magnetic resonance imaging
SMN: sensory motor network
SN: salience network
TPN: task positive network
VAN: ventral attention network.
