# Supplementary material for: A comprehensive assessment of resting state networks: bidirectional modification of functional integrity in cerebro-cerebellar networks in dementia
Source: Front Neurosci. 2014 Jul 30;8:223. doi: 10.3389/fnins.2014.00223 (PMC4115623; doi:10.3389/fnins.2014.00223)
Supplement: Supplementary file 1 [file DataSheet1.DOC]

| **Reference Study** | **RSNs identification method** | **Main Findings** |
| --- | --- | --- |
| *Minati et al. 2014* | Rosazza et al. 2011 | Alterations in the DMN in amnestic MCI (a-MCI). |
| *Agosta et al., 2012* | Beckmann at al .2005;  Smith et al. 2009 | Alterations in the DMN and in the FCN in Alzheimer disease (AD) |
| *Binnewijzend et al., 2012* | Beckmann at al .2005;  Smith et al. 2009 | Alterations in the DMN, visual cortex, basal ganglia and cerebellum network. |
| *Bai et al .2012* | Greicius et al. 2007  Van de Ven et al. 2004, 2008 | Abnormal relationship detected between the DMN, the Auditory Network (AN) and the Self Referential Network (SRN), in amnestic MCI (a-MCI) |
| *Brier et al. 2012* | Shulman et al., 1997;  Raichle et al., 2001;  Greicius et al., 2004; Seeley et al., 2007;  Zhou et al., 2010;  Sestieri et al., 2011 | Functional connectivity alterations shown in large scalenetworks, including the DMN, dorsal attention network (DAN), executive control network (ECN), salience network (SN), and sensory motor network (SMN) in AD.  rs-fMRI abnormalities increase with disease progression. |
| *Jones et al., 2011* | Visual inspection | Alterations in the DMN in AD. |
| *Petrella et al., 2011* | RSN templates supplied in GIFT tool* | Alterations in the DNM increased with the disease progression in AD. |

**Supplemetary Table**

Summary of cited literature on FC alterations in AD and MCI. Because literature on this topic is huge we report the key points of some of the most recent rs-fMRI studies on AD.

* Group ICA of fMRI Toolbox (GIFT) tool, [*http://icatb.sourceforge.net/gift/gift_startup.php*](http://icatb.sourceforge.net/gift/gift_startup.php)
